# Comparative Analysis of Proteomics and Transcriptomics during Fertility Transition in a Two-Line Hybrid Rice Line Wuxiang S

**DOI:** 10.3390/ijms20184542

**Published:** 2019-09-13

**Authors:** Hao Chen, Jing Jin, Hongyuan Zhang, Ying Wang, Qian Li, Yu Zou, Xingguo Huang, Baojin Zhou, Ruo Zhou, Yi Ding

**Affiliations:** 1 State Key Laboratory of Hybrid Rice, Department of Genetics, College of Life Sciences, Wuhan University, Wuhan 430072, China; haochen2013@whu.edu.cn (H.C.); jinjing1130@whu.edu.cn (J.J.); yingwang@whu.edu.cn (Y.W.); 2017202040074@whu.edu.cn (Q.L.); zouxiaoyu@whu.edu.cn (Y.Z.); 2 Institute of Vegetable, Wuhan Academy of Agricultural Sciences, Wuhan 430065, China; hongyuanzhang2011@163.com; 3 Wuhan Wuda Tianyuau Bio-Tech Co., Ltd., Wuhan 430070, China; huangxingguo1122@126.com; 4 Deepxomics Co., Ltd., Shenzhen 518000, China; zhoubaojin@deepxomics.com (B.Z.); zhouruo@deepxomics.com (R.Z.)

**Keywords:** rice (*Oryza sativa* L.), male sterile line, iTRAQ, RNA-seq, differentially abundant proteins

## Abstract

The two-line hybrid rice is an important factor of a global crop, but its fertility transition mechanism is unclear. Here, a comparative proteomics and transcriptomics analysis was completed on the two-line hybrid rice line Wuxiang S (WXS) to explore its molecular mechanism and protein regulation during fertility transition. A total of 340 differentially abundant proteins (DAPs) were identified using iTRAQ between the pollen mother cell formation stage (P2) and the meiosis stage (P3). There were 3541 and 4247 differentially expressed genes (DEGs) in P2 and P3 between WXS (Sterile, S)-WXS(S) and WXS (Fertile, F)-WXS(F), respectively, of which 92 and 71 DEGs had corresponding DAPs. Among the DAPs and DEGs, 65 (SP2 vs. FP2) and 55 (SP3 vs. FP3) corresponding DEGs and DAPs (cor-DEGs-DAPs) showed the same expression trend, indicating the cor-DEGs-DAPs genes might play vital roles in WXS fertility transition. Further analysis indicated that cor-DEGs-DAPs proteins were related to energy metabolism-related proteins in anther development and were accompanied by the activation of the stress response pathway and modifications to the cell wall, which ultimately affected the fertility transition of the PTGMS rice line WXS.

## 1. Introduction

Rice (*Oryza sativa* L.) plays a crucial role in supporting crops to half of the population all over the world [1]. The technologies of three- and two-line hybrid rice have greatly increased rice production. According to the genetic basis, male sterility can be classified as cytoplasmic male sterility (CMS) and genic male sterility (GMS). Usually, CMS can be restored by nuclear restorer gene(s), providing the genetic basis for the development of the three-line hybrid rice consisting of a male sterile line, a maintainer line, and a restorer line. Many GMS mutations have been found to be regulated by environmental conditions, such as photoperiod, temperature, or both, and thus, they are referred to as photoperiod-sensitive genic male sterility (PGMS) and temperature-sensitive genic male sterility (TGMS). These characteristics have provided opportunities to develop two-line hybrid rice, since the male sterile line can be used for hybrid seed production under restrictive conditions and propagate itself under permissive conditions [2]. Moreover, two-line hybrid rice cultivation is more effective than three-line hybrid rice, because it produces a higher yield and can cross freely in comparison [3,4]. The photo-thermosensitive genic male sterile (PTGMS) rice line is an important component of the two-line hybrid rice system. The fertility transition of the two-line hybrid rice male sterile line depends on environmental conditions (temperature and day length). Most PTGMS rice lines exhibit male sterility at long day lengths and high temperatures during the anther development, and exhibit male fertility at short day lengths and lower temperatures [5,6]. Therefore, the male sterile line of PTGMS rice can be used as a maintainer line under short day lengths and lower temperatures. The fertility transition from the pollen mother cell (PMC) formation stage to the meiotic division stage in two-line hybrid rice is a critical period [2,7]. The photoperiod-sensitive genic male sterile (PGMS) and the temperature-sensitive genic male sterile (TGMS) rice are two major types of PTGMS germplasm resources. NK58S was one of the first PGMS rice found in China [8]. Pollen development was aborted in NK58S from the PMC formation stage to the entire process of pollen development under long-day conditions, which also influenced the development of tapetum [9]. The abnormal tapetum could not support enough nutrients for the developing microspores, and the premature tapetum of NK58S was degenerated in programmed cell death (PCD) earlier than NK58 [10]. AnS-1 was one of the TGMS rice that was found to have wrinkled abortive pollen grains under high temperatures. In the PMC formation and meiosis stages, the microspore mother cells of AnS-1 could undergo normal meiosis [11].

Male sterility is a prevalent phenomenon in flowering plants, so it is important to understand the underlying molecular mechanisms to improve the production of crops. Several comparative proteomics of the anther development between wild type and male sterile plants have been reported in rice, cotton, and tomato [12,13,14,15]. By using the technology of 2-DE proteomics, the young panicle proteins of the TGMS rice Zhu-1S during different developmental stages were compared under sterile and fertile conditions. Most of the 20 proteins identified were associated with energy metabolism, protein biosynthesis, cell wall formation, and stress responses [12]. In addition, 83 identified proteins were found in the young panicles of the TGMS rice lines Zhu-1S and Zhun S at the fertility alternation sensitivity stage. Compared to Zhun S, Zhu-1S possessed lower ROS (reactive oxygen species)-scavenging, indole-3-acetic acid level, soluble protein, sugar contents, and faster anther wall disintegration [15]. Two proteomes of PGMS cotton under sterile and fertile conditions were also reported as a result of using the 2-DE proteomics technique and iTRAQ, and many differentially abundant proteins were found to be involved in exine formation, protein metabolism, calcium ion binding, etc. [13,16]. Compared to the 2-DE technique, iTRAQ can detect extremely small and acidic proteins [17,18]. All of the proteomic studies could advance our understanding of the molecular mechanisms of fertility transition in PTGMS plants. However, until now, the genetic and molecular mechanisms of fertility transition in PTGMS rice were unclear.

In the present study, we used a novel PTGMS line, Wuxiang S (WXS), which was derived from a mutant *tms*5 locus in *indica* rice as an experiment material to explore the mechanism of the fertility transition of PTGMS rice lines. The young plant panicles were compared between WXS (Sterile, S)-WXS(S) and WXS (Fertile, F)-WXS(F) under fertility transition using iTRAQ and RNA-seq. Then, by combining the results of iTRAQ analysis and our differential transcriptomic results, we found that several corresponding DEGs and DAPs (cor-DEGs-DAPs) were involved in key pathways associated with fertility transition. This result may broaden our further understanding of the mechanisms of fertility transition in the PTGMS rice line.

## 2. Results

### 2.1. Cytological Characteristics of WXS during Fertility Transition

**Figure 1:**
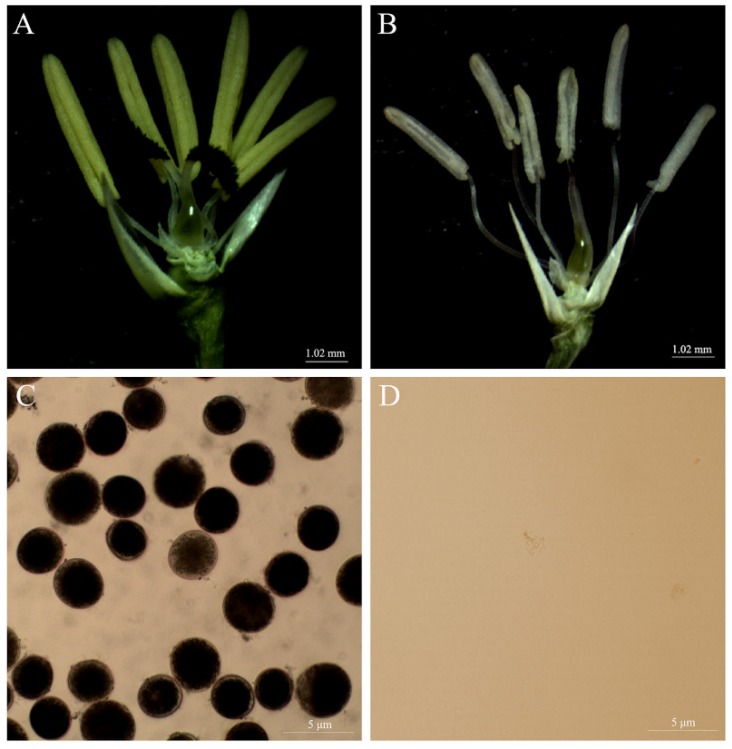
Cytological observation of pollen morphology. (**A**) The anthers of WXS (F) were yellow and full under the low temperature (~22 °C) and shorter day lengths, and (**C**) the pollen grains of WXS (F) were deeply stained by iodine-potassium iodide (I_2_-KI). However, (**B**) the anthers of WXS (S) were white and shriveled under higher temperatures (> 23 °C) and longer day lengths, and (**D**) the pollen grains of the WXS(S) were completely absent.

To determine the critical condition of WXS fertility conversion, we performed cytological observations, and the results showed that the anthers of WXS (F) were yellow and full under normal pollen development (Figure 1A), and the pollen grains were deeply stained by iodine-potassium iodide (I_2_-KI) under the low temperature (~22 °C) and shorter day lengths (Figure 1C). However, the anthers of WXS (S) were white and shriveled under abnormal pollen development (Figure 1B), and the pollen grains were completely absent at higher temperatures and longer day lengths in the same developmental period (Figure 1D). Moreover, WXS was chosen as an ideal and reliable material for our study of fertility transition of two-line hybrid rice.

### 2.2. Overview of Quantitative Proteomics Analysis

The experimental strategy is shown in Figure S1. A total of 1,609,680 spectra were identified by iTRAQ-LC-MS/MS proteomic analysis in the present study. After the data were filtered, a total of 340,777 unique spectra and 63,227 unique peptides were obtained. To avoid identification errors, 8146 proteins with FDR (false discovery rate) ≤ 0.01 were further confirmed using the Rice Genome Annotation Project database (http://rice.plantbiology.msu.edu/). Then, 5671 (repetition 1), 6470 (repetition 2), and 6005 (repetition 3) proteins were respectively identified (Table S1), all identified proteins’ annotations are shown in Table S2 and 4546 of the proteins were shared in the three biological replicates (Figure S2). The distribution of protein mass showed that about 75% of the identified protein mass was 10 to 70 kDa (Figure S3A). The peptide length of the identified proteins shows that most proteins contain less than 10 peptides, and about 14% of proteins contain more than 10 peptides (Figure S3B). Approximately 90% of the proteins included at least two peptides (Figure S3C).

For protein orthologous classification, all identified proteins were compared with the Cluster of Orthologous Groups (COG) database to predict the possible protein function. The annotated proteins were classified into 25 clusters (Figure S4). In the COG results, the largest cluster was the ‘translation, ribosomal structure, and biogenesis’ that included 471 proteins, followed by ‘general function prediction only’, and ‘posttranslational modification, protein turnover, chaperones’. The smallest cluster was relevant to ‘RNA processing and modification’, which are only six proteins.

Differentially expressed proteins (DAPs) were defined as proteins with an FC > 1.2 and *p* < 0.05. In the present study, DAPs were identified at least twice in three biological replicates and had the same change trend. Based on these criteria, we found that 119 DAPs were upregulated, and 144 DAPs were downregulated in SP2 vs. FP2, and 87 DAPs were upregulated, and 162 DAPs were downregulated in SP3 vs. FP3 (Figure S5, Table S3).

The DAPs were searched in the gene ontology (GO) database. Our results showed that the most significantly enriched GO term in the biological process was the ‘single-organism metabolic process’, which had 84 proteins, followed by a ‘response to stimulus’, which possessed 51 proteins. In the molecular function, DAPs that were enriched in ‘oxidoreductase activity’ had 44 proteins, and ‘cation binding’ had 61 proteins. In the cellular component, ‘cell periphery’, ‘extracellular region’, and ‘cell wall’ were the most enriched GO terms, among which there were 37, 24, and 19 proteins, respectively (Figure S6).

Kyoto Encyclopedia of Genes and Genomes (KEGG) analysis also revealed that the carbohydrate metabolism pathway was one of the most significantly enriched pathways, with 45 proteins; followed by amino acid metabolism (26 proteins + 15 proteins); and folding, sorting, and degradation (26 proteins). In genetic information processing and metabolism, few DAPs were involved in signal transduction, environmental adaptation, and energy metabolism. These results showed that these DAPs might be influenced by the environment and were further involved in metabolic pathways (Figure S7, Table S4).

### 2.3. Transcriptional Analysis of Differentially Expressed Genes (DEGs)

The rice young panicles of WXS (S) and WXS (F) at the P2 and P3 stages (SP2, FP2, SP3, FP3) were also analyzed using RNA-seq with three biological replications, which in total generated ∼1.4 billion raw sequence reads, and then the unique mapped reads of each sample was an average of 83.58% (Table S5). A total of 47,919 genes in all young panicle samples were identified by the transcriptome and there were 3541 (SP2 vs. FP2) and 4247 (SP3 vs. FP3) differentially expressed genes (DEGs, fold-change >2 with a *p*-value < 0.05), respectively.

### 2.4. Comparison Analysis Between the Transcriptome and Proteome Data

The combination analysis of transcriptomic and proteomic data showed that a total of 7498 proteins corresponded with their transcriptomic mRNAs in our study. These results showed that the correlation of the proteins with their corresponding transcripts (mRNAs) were 0.095 in SP2 vs. FP2 and 0.184 in SP3 vs. FP3, respectively. However, there was a high correlation between DAPs and their corresponding transcripts, with 0.468 in SP2 vs. FP2 and 0.376 in SP3 vs. FP3 (Figure S8), respectively.

Our analysis results showed that, altogether, there were 3541 and 4247 differentially expressed genes (DEGs) in P2 and P3 between WXS(S) and WXS(F), respectively, of which 92 and 71 DEGs had corresponding DAPs (Figure S9). The paired DEGs and corresponding DAPS were named cor-DEGs-DAPs genes. We also compared the DAPs and DEGs between P2 and P3 in WXS(S) and WXS(F), respectively, there were correspondingly 8 cor-DEGs-DAPs (Figure S9A), and 11 cor-DEGs-DAPs (Figure S9B). They showed that these two comparison groups have few differences. The cor-DEGs-DAPs genes were enriched in SP2 vs. FP2 and SP3 vs. FP3. Among the cor-DEGs-DAPs genes, 65 (SP2 vs. FP2) and 55 (SP3 vs. FP3) genes showed the same expression trend (Table S6). So, we suggest tthat these cor-DEGs-DAPs genes might play vital roles during the fertility transition stages of the PTGMS rice line WXS.

### 2.5. GO and Pathway Enrichment Analysis of the cor-DEGs-DAPs Genes

Our results of the GO terms’ enrichment analysis for cor-DEGs-DAPs genes showed that the proportion of ‘cell’ and ‘cell part’ in the cell components were the two largest subcategories, and among the two parts were 39 and 39 genes in SP2 vs. FP2, respectively, and 31 and 30 genes in SP3 vs. FP3, respectively. In the molecular function, the number of genes involved in the ‘binding activity’ and in the ‘catalytic activity’ were the highest: 37 and 35 genes in SP2 vs. FP2, 30 and 43 genes in SP3 vs. FP3, respectively. In the biological process, the number of genes involved in the ‘cellular process’ and in the ‘metabolic process’ possessed the two largest proportions, 39 and 37 genes in SP2 vs. FP2, and 33 and 41 genes in SP3 vs. FP3, respectively (Figure S10).

**Figure 2:**
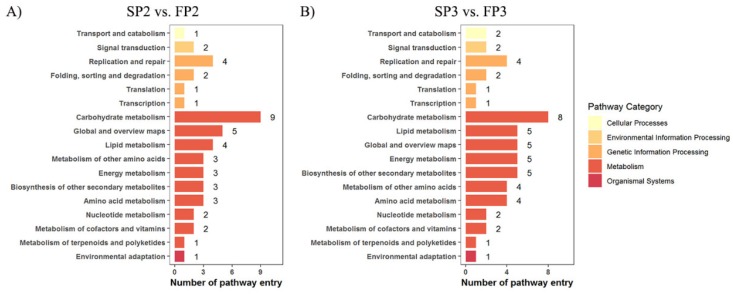
KEGG pathway analysis of cor-DEGs-DAPs genes in SP2/FP2 (**A**) and SP3/FP3 (**B**).

The pathway analysis of cor-DEGs-DAPs genes showed that the main enrichment pathways were ‘metabolic pathway’ (ko01100), ‘biosynthesis of secondary metabolites’ (ko01110), ‘protein processing in endoplasmic reticulum’ (ko04141), and ‘phenylpropanoid biosynthesis’ (ko00940) in SP2 vs. FP2. The main enrichment in the ‘metabolic pathway’ (ko01100), the ‘biosynthesis of secondary metabolites’ (ko01110), ‘phenylpropanoid biosynthesis’ (ko00940), and ‘starch and sucrose metabolism’ (ko00500) occurred in SP3 vs. FP3. Moreover, the KEGG pathways of the cor-DEGs-DAPs genes were significantly enriched in ‘photosynthesis’, ‘oxidative phosphorylation’, and ‘plant hormone signal transduction’, which suggested that these processes could take part in regulating WXS fertility transition (Figure 2, Table S7).

### 2.6. The cor-DEGs-DAPs Genes Enriched in the Cell Wall of the GO Term

Our analysis result of the GO function that was enriched for the cor-DEGs-DAPs genes showed that some genes were involved in cell wall development pathways (Figure S11, Table 1). These genes, including *LOC_Os06g48180.1* (glycosyl hydrolases family 16, putative, expressed, GH16); *LOC_Os03g48770.1* (Cupin domain containing protein, expressed); *LOC_Os02g32980.1* (Cupin domain containing protein, expressed); *LOC_Os01g24680.1* (3-hydroxyacyl-CoA dehydrogenase, putative, expressed); *LOC_Os05g04380.1* (peroxidase precursor, putative, expressed); *LOC_Os06g43640.1* (Ser/Thr protein phosphatase family protein, putative, expressed); and *LOC_Os01g18170.1* (Cupin domain containing protein, expressed), participated in the cell wall synthesis pathway in the cellular component (GO) (the green box in Figure S11A) and in the biological process (GO) (the green box in Figure S11B). In addition, the expression level of these genes was lower in WXS (S) than in WXS (F). Therefore, we suggest that these genes might be associated with pollen abortion in WXS (S).

### 2.7. The Protein–Protein Interaction Network of the cor-DEGs-DAPs Proteins

**Figure 3:**
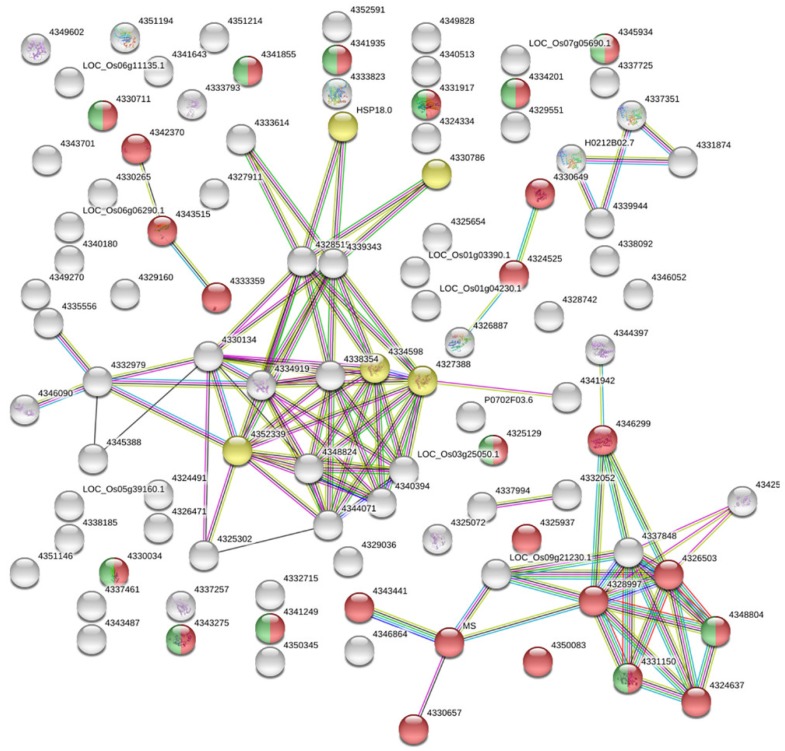
The protein–protein interaction network of the cor-DEGs-DAPs protein analyzed by STRING software. The red circles represent ‘metabolic pathways’; the green circles represent ‘biosynthesis of secondary metabolites’; and the yellow circles represent ‘protein processing in endoplasmic reticulum’. The genes are color-coded by their clustering, as determined by the MCL (Markov Clustering algorithm) clustering inflation parameter of 3. The cor-DEGs-DAPs proteins are represented by node, whereas different colored lines represent different evidences for the predicted functional relationship between proteins; red line: gene fusion evidence; dark blue line: co-occurrence evidence; black line: co-expression evidence; yellow line: text-mining evidence; green line: neighborhood genome evidence; light blue line: database evidence; and pink line: experimental evidence.

To further clarify the inter-connections among the cor-DEGs-DAPs genes, a STRING (Search Tool for the Retrieval of Interacting Genes/Proteins) analysis was performed to find that the cor-DEGs-DAPs were most enriched in metabolic pathways (the red circles in Figure 3) and in the biosynthesis of secondary metabolites (the green circles in Figure 3). Moreover, we found that a number of heat shock proteins were highly clustered in protein processing in the endoplasmic reticulum (the yellow circles in Figure 3). These proteins might respond to the environmental condition of high temperature in WXS during the fertility transition stages.

### 2.8. The cor-DEGs-DAPs Genes Examined by qPCR

**Figure 4:**
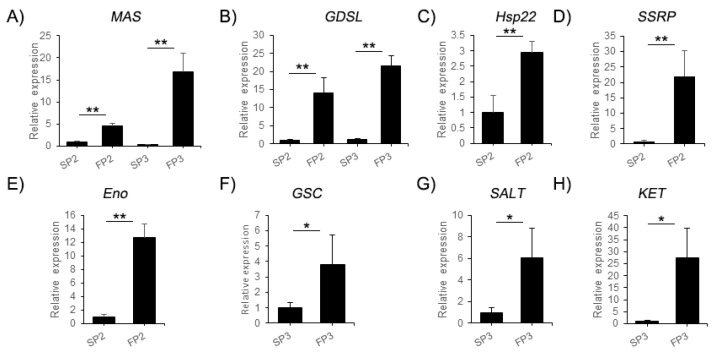
Differential expression levels of eight cor-DEGs-DAPs genes validated by quantitative real-time PCR. The candidate genes were (**A**) *MAS (LOC_Os04g40990.1)*, (**B**) *GDSL (LOC_Os05g11910.1)*, (**C**) *Hsp22 (LOC_Os02g52150.2)*, (**D**) *SSRP (LOC_Os05g08970.1)*, (**E**) *Eno (LOC_Os01g54860.1)*, (**F**) *GSC (LOC_Os02g50240.1)*, (**G**) *SALT (LOC_Os02g18410.1)* and (**H**) *KET (LOC_Os02g57260.1)*. The error bars indicate standard deviation of three replicates. Asterisks indicate a significant difference as determined by student’s *t*-test (* *p* < 0.05; ** *p* < 0.01).

To further validate the correspondence between the transcript level of mRNA and protein expression levels, eight cor-DEGs-DEPs genes were validated by quantitative real-time PCR. The candidate genes were *MAS (LOC_Os04g40990.1)*, *GDSL (LOC_Os05g11910.1)*, *Hsp22 (LOC_Os02g52150.2)*, *SSRP (LOC_Os05g08970.1)*, *Eno (LOC_Os01g54860.1)*, *GSC (LOC_Os02g50240.1)*, *SALT (LOC_Os02g18410.1)* and *KET (LOC_Os02g57260.1)* (Figure 4). In addition, the same expression trend as that of the proteins was verified by iTRAQ, which proved that the iTRAQ data were reliable.

## 3. Discussion

In the present study, we combined the iTRAQ proteomics results and our differential transcriptomics results to analyze the dynamic profiles of protein regulation and gene expression in young rice panicles during fertility transition stages. Our results showed that the fertility transition of the PTGMS rice line WXS deals with plant hormone signal transduction, protein processing in the endoplasmic reticulum, starch and sucrose metabolism, phenylpropanoid biosynthesis, cutin, suberine, and wax biosynthesis, etc. Therefore, it is a complicated network regulation.

### 3.1. Plant Hormone Signal Transduction

Under different environments, WXS rice sense environmental signals and transmit them to the cells that produce plant hormones to adapt to stress. In the study, in SP2 vs. FP2 and SP3 vs. FP3, the gene *LOC_Os06g48180.1* (Glycosyl hydrolases family 16, putative, expressed, GH16) was found, for which the rates of SP2/FP2 and SP3/FP3 were 0.51 and 0.54, respectively, which showed that the *GH16* had risen in WXS (F). Furthermore, the glycosyl hydrolase genes were divided into 29 families, and the majority of these hydrolases were involved in cell wall metabolism [19]. For example, in *Arabidopsis thaliana*, the *GH* family may participate in loosening the cell wall to ensure pollen dilatation after mitosis and may participate in cell expansion during pollen germination. In addition, it was reported that *GH1* may be involved in the metabolic process of the cell wall in rice [20]. In this study, the expression level of *GH16* in the critical period of WXS pollen development was found to be lower in WXS(S) than in WXS (F), and it was found in the GO pathway of the cell wall. Therefore, we suggest that *GH16* might participate in the process of anther cell wall development and in the absence of pollen in WXS(S) rice by hormone signal transduction.

### 3.2. Protein Processing in Endoplasmic Reticulum

We found that eight cor-DEGs-DAPs genes were involved in protein processing in endoplasmic reticulum (Table 2, Figure S12) in our study. These genes, which included *LOC_Os01g08860.1* (hsp20/alpha crystallin family protein, putative, expressed); *LOC_Os02g52150.2* (heat shock 22 kDa protein, mitochondrial precursor, putative, expressed); *LOC_Os03g60620.1; LOC_Os05g23740.1; LOC_Os05g38530.1* (DnaK family protein, putative, expressed); *LOC_Os04g01740.1; LOC_Os12g32986.1* (heat shock protein, putative, expressed) and *LOC_Os03g45340.1* (hsp20/alpha crystallin family protein, putative, expressed), were connected with the heat shock protein (Hsps). In this study, they had higher expressions in WXS (S) than in WXS (F). Hsps are a class of functionally related proteins that are involved in the folding and unfolding of other proteins. Calreticulin, calnexin, and BiP are the key components of the ER protein folding machinery, and they exist throughout microspore and pollen development [21,22]. It was also reported that Hsps existed in different stages of anther development in different plants, such as *Arabidopsis*, tobacco, and tomato [23,24]. In *Arabidopsis*, the Hsp40 protein TMS1 participated in pollen tube growth under a heat shock environment [25]. The luminal binding protein (BiP) is the chaperone of the Hsp70/DnaJ and Hsp90 proteins. Moreover, the unfolded protein response (UPR) could produce several mutants in the pathway of pollen development and result in male gametophyte defective or lethal phenotypes [26]. After a heat shock or long-term exposure to mild heat, the UPR is upregulated in male reproductive tissues at the transcript and protein level, which suggests that the UPR has a function in the acclimation of developing pollen to heat [23,27,28]. In this study, the Hsps had a higher expression in WXS (S) than in WXS (F), which might have produced more misfolded proteins that could result in pollen abortion.

### 3.3. Starch and Sucrose Metabolism

In this study, we found gene *LOC_Os07g 35480.3* (glucan endo-1,3-beta-glucosidase precursor, putative, expressed) in SP2 vs. FP2 and in SP3 vs. FP3, and their expression ratios were 0.68 and 0.62, respectively; we also found the gene *LOC_Os03g45340.1* (hsp20/alpha crystallin family protein, putative, expressed) in SP3 vs. FP3, and the ratio was 0.74. These genes affect the synthesis of D-glucose and amylose, respectively (Figure S13). As shown in Figure S13, amylose could affect the synthesis of starch and glycogen. During early anther development, large amounts of sugars are needed to support the anther development, and at later stages of pollen maturation, plenty of starch should be accumulated [29]. The disordered metabolism of starch and sucrose during anther development may lead to rice sterility [30]. Starch abundance is also critically required for pollen vigor [31,32,33]. Therefore, we suggested that the increased expression of these genes contributes to energy supply and affects the development of anther.

### 3.4. Phenylpropanoid Biosynthesis

The metabolism of phenylpropanoids is very important for the formation of the cell wall in pollen grains during pollen development [34]. The phenylpropanoid biosynthesis pathway is connected to the biosynthesis of flavonoids [35]. Moreover, flavonoids are essential for pollen development in many species [36,37]. Deamination of phenylalanine (Phe) by phenylalanine ammonia-lyase (PAL) is the first step of phenylpropanoid biosynthesis. It was reported that PAL cDNA in the tobacco tapetum tissue could induce partial male sterility [38]. In this study, we found the gene *LOC_Os 02g41630.2* (phenylalanine ammonia-lyase, putative, expressed) in SP2 vs. FP2 and in SP3 vs. FP3, and their expression ratios were 0.78 and 0.68, respectively. *PAL* had high expression in the fertile line WXS (F), which suggested that the low expression of the *PAL* may lead to the absence of the pollen in sterility line WXS (S).

### 3.5. Cutin, Suberine, and Wax Biosynthesis

The cuticle has a vital function to male reproductive development in rice [39,40]. The cutin and waxes constitute the functionally lipophilic portion in the cuticle [41]. It was shown that cutin, suberine, and wax biosynthesis may be involved in unsaturated fatty acid and fatty acid elongation (Figure S14) in our KEGG analysis. *HOTHEAD* is involved in the biosynthesis of unsaturated fatty acids in *Arabidopsis thaliana* [42]. Additionally, *HOTHEAD* is necessity for pollen fertility in rice [43]. We found the gene *LOC_Os09g19930.1* (HOTHEAD precursor, putative, expressed) in SP2 vs. FP2, and its expression ratios was 1.48, which suggests that it may be involved in pollen development.

### 3.6. Proposed Model for Gene Transcript Regulation of PTGMS Rice during the Fertility Transition Stage

**Figure 5:**
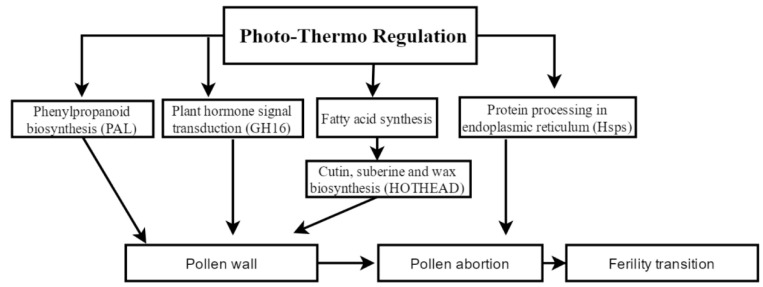
The proposed model of fertility transition mechanism in the PTGMS rice line WXS. Hsps, heat shock proteins; *GH16*, glycosyl hydrolases family 16; *PAL*, phenylalanine ammonia-lyase.

Based on our analysis results and the functions and expression patterns of the cor-DAPs-DPGs proteins, and the above discussion, we proposed a model that might explain the fertility transition in PTGMS rice (Figure 5) in the present study. Initially, the heat shock proteins (Hsps) in the anther responded to the high environmental temperature. Then, the Hsps were involved in the folding and unfolding of other proteins. With the growth and development of rice plants, the Hsps expression was higher in WXS (S) than in the (F), and this result might produce more misfolded proteins that could be involved in pollen abortion. At the same time, plant hormone signal transduction (GH16) was involved in the development of the anther cell wall, and the cuticle is the important part of the pollen wall, and is involved in phenylpropanoid biosynthesis pathways, and biosynthesis of fatty acid. In phenylpropanoid biosynthesis, *PAL* is a key upstream gene, it has low expression in WXS (S). In fatty acid biosynthesis, 3-hydroxyacyl-CoA dehydrogenase had lower expressions in WXS (S) than in WXS (F). Additionally, in cutin, suberine, and wax biosynthesis, the HOTHEAD precursor had high expression in WXS (S), which could result in the abortion of the pollen in WXS (S). Moreover, the metabolic pathway and starch and sucrose metabolism were all involved in the development process and energy supply. In WXS (S), they might not be able to offer enough energy to support anther development. Finally, the regulatory interactions described above might lead to fertility transition in the PTGMS rice line WXS.

## 4. Materials and Methods

### 4.1. Plant Materials

The two-line hybrid rice line WXS was cultivated in Wuhan and Hainan of China. Between January and April 2017, the plants were grown in the natural ecological experimental field of Guang-Po-Zhen (18°57′ N, 110°05′ E), Hainan province of China. During this period, the average temperature of Hainan was less than 22 °C. The plants were male fertile plants that were designated as WXS (F). From July to August 2017, the WXS plants were grown in a rice paddy at the Institute of Genetics of Wuhan University (30°54′ N, 114°37′ E), Wuhan, Hubei province of China, under natural conditions, with daily average temperatures that were higher than 24 °C and average day lengths that were longer than 12 h. The higher temperatures and longer day lengths could induce WXS sterility, and these plants were designated as WXS (S). We sowed the seeds every seven days and harvested the young panicles respectively. All of the plants’ young panicles were harvested from the natural ecological experimental field in Wuhan and Hainan during the PMC formation stage (P2) and the meiosis stage of PMC (P3). Correspondingly, they were designated as SP2, SP3, FP2, and FP3. Twelve samples (three biological replications for each stage) were conducted. After the sample materials were harvested, they were frozen in liquid nitrogen and kept at −80 °C. The mature pollens and pollen grains were dyed with 1% potassium iodide solution (I_2_-KI) to identify pollen fertility.

### 4.2. Protein Preparation

Protein extraction was carried out according to the method reported in our laboratory [44]. The procedure was as follows: 0.4 g young rice panicles were ground into powder with liquid nitrogen, then extracted with 1 mL RIPA buffer (50 mmol/L Tris-HCl pH 7.4, 100 mmol/L NaCl, 1 mmol/L PMSF, 1 mmol/L EDTA, 1% NP-40, 1% sodium deoxycholate, 2% SDS) and centrifuged at 35,000× *g* for 20 min. Then, each 1 mL supernatant was mixed well with 4 mL methanol, 1 mL chloroform, and 3 mL water, and then was centrifuged at 14,000× *g* for 2 min. The supernatant was carefully discarded, added with 2 mL methanol, was vortexed, and then finally centrifuged at 14,000× *g* for 2 min. The deposit was dried, 400 μL buffer (200 mmol/L Tris–Cl, pH 8.0, 8 mol/L urea, 4 mmol/L CaCl_2_) was added, and then stored in a −80 °C refrigerator. Three biological replicates were conducted.

### 4.3. Protein Digestion

The protein extract was added to 2 mL 9 mol/L urea, then underwent ultrasonication disintegration. Subsequently, it was centrifuged for 20 min at 12,000× *g*, per 1 mL supernatant was added to DTT to a final concentration of 10 mmol/L, and then it was incubated at 37 °C for 1 h, followed by the addition of iodoacetamide alkylation at a final concentration of 40 mmol/L at 25 °C in the dark for 30 min. Then, 500 μg quantified protein (Promega, Madison, WI, USA) was added to 10 μL trypsin (a final enzyme at a substrate ratio of 1:50), and finally, the digests were incubated at 37 °C overnight and were dried by vacuum centrifugation.

### 4.4. iTRAQ Labeling

After digestion, the peptides were added to 20 μL dissolution buffer, mixed well, and subsequently centrifuged for 5 min at 12,000× *g*. Then, one unit of the iTRAQ reagent was reconstituted in 50 μL isopropanol, and the peptide samples FP2, SP2, FP3, and SP3 that were obtained from WXS (S) and WXS (F) were labelled with the iTRAQ reagents 113, 114, 115, and 116, respectively (Applied Biosystems, Framingham, MA, USA) according to the manufacturer’s instructions. Then, the mixed samples were labelled with 121 tags. The samples were labelled with three biological replicates. These peptides were incubated at room temperature for 2 h; then, they were pooled and dried by vacuum centrifugation.

### 4.5. High pH Reverse Phase Fractionation

High pH reverse phase fractionation chromatography was performed with an Agilent 1260 HPLC system (Agilent, Waldbronn, Germany). The iTRAQ-labeled peptides were resolved in buffer A (20 mmol/L HCOONH_4_ in ammonia, pH 10) and were loaded onto a 4.6 × 150 mm C18 column that contained 5-μm particles (TechMate, C18-ST). The peptides were eluted at a 0.2 mL/min flow rate with buffer B (20 mmol/L HCOONH4 in 80% ACN and an equal quantity of 25% ammonia water was added) using a gradient of 0–5% for 5 min, 5–25% for 20 min, 25%–45% for 15 min, 45%–90% for 5 min, 90%–95% for 1 min, and 95%–100% for 14 min. The elution was monitored based on a wavelength of 216 nm to make sure equal quantity peptides were collected from each pool, and the fractions were collected in 1-min intervals. The fractions were combined into 15 pools and were desalted on C18 cartridges.

### 4.6. RPLC-ESI-MS/MS Analysis Based on Triple TOF 5600

RPLC-ESI-MS/MS was used to detect the samples. LC-MS/MS detection was carried out according to the previous method [45] on a hybrid quadrupole-TOF LC/MS/MS mass spectrometer (Triple TOF 5600^+^, AB Sciex) that was equipped with a nanospray source. First, the peptides were loaded onto a C18 trap column (5 µm, 5 × 0.3 mm, Agilent Technologies); then, they were eluted into a C18 analytical column (75 μm × 150 mm, 3 μm particle size, 100 Å pore size, Eksigent). Mobile phase A (3% DMSO, 97% H_2_O, 0.1% formic acid) and mobile phase B (3% DMSO, 97% ACN, 0.1% formic acid) were used to build a 30-min gradient that consisted of 0 min at 5% B, 15 min at 5%–35% B, and 1 min at 35%–80% B. In addition, the gradient was maintained at 80% B for 5 min, followed by 0.1 min at 80%–5% B, with a final step at 5% B for 8.9 min. A constant flow rate was set at 300 nL/min. MS scans were conducted from 350 to 1500 amu, with a 250-ms time span. For MS/MS analysis, each scan cycle consisted of one full-scan mass spectrum (with m/z ranging from 350 to 1500 and charge states ranging from 2 to 5) followed by 40 MS/MS events. The threshold count was set to 120 to activate MS/MS accumulation, and a former target ion exclusion was set for 18 s.

### 4.7. iTRAQ Data Analysis

The raw data files were converted into Mascot Generic Format (.mgf) files using AB SCIEX MS Data Converter 1.3 beta software. The MGF files were searched three search engines (MyriMatch v2.2.8634, X!Tandem v2015.04.01.1, and MS-GF+ v2016.06.29) through IPeak against a rice protein database (Release 7, Rice Genome Annotation Project). The MS/MS searching parameters included a precursor mass tolerance of 20 ppm, a fragment ion mass tolerance of 0.05 Da, full cleavage by trypsin with one missed cleavages permitted, Carbamidomethyl (C), iTRAQ 8 plex (K) and iTRAQ 8 plex (N-term) as the fixed modification, and Oxidation (M) and iTRAQ 8 plex (Y) as variable modifications. The false discovery rate (FDR) at both peptide spectrum matching (PSM) and protein levels was set ≤ 1%. Proteins with at least one unique spectrum were used for quantification and only a unique peptide was used for protein quantification. After MS/MS searching, IQuant [46,47] was used for protein quantification with VSN normalization.

Furthermore, to identify differentially abundant proteins (DAPs), the proteins had a fold change (FC) > 1.2 (*p* < 0.05, Student’s *t*-test) and needed to be identified at least twice in three biological repetitions. DAPs in SP vs. FP were combined with DAPs in SP2 vs. FP2 and in SP3 vs. FP3. To identify the significant DAPs between WXS (S) and WXS (F) in the same developmental stage, we defined the proteins with an FC > 1.2 and *p* < 0.05 as DAPs. Data are available via ProteomeXchange with identifier PXD.

### 4.8. Protein Annotation and Classification

In the GO and KEGG pathway enrichment analysis of DAPs and cor-DAPs-DEGs, we compared the significant DAPs to all of the identified proteins, as a background, and the formula used in these analyses was as follows:

$$\text{P}=1-\sum_{i=0}^{m-1}\frac{\left(\begin{array}{c} M\\ i \end{array}\right)\left(\begin{array}{c} N-M\\ n-i \end{array}\right)}{\left(\begin{array}{c} N\\ n \end{array}\right)}$$

where N is the number of GO/KEGG pathway entries in all identified proteins; n is the number of GO/KEGG pathway entries that represent significant differentially abundant proteins; M is the number of GO/KEGG pathway entries that can be matched to all identified proteins; and m is the number of GO/KEGG pathway entries that can be matched to a significant differentially expressed protein. If the *p*-value of the hypergeometric test was less than 0.05, the differential protein was significantly enriched in the GO/KEGG pathway entry.

### 4.9. Protein Interaction Analysis Using STRING

Gene lists were analyzed using the STRING database to identify the predicted interactions of proteins. The network connections were visualized by their confidence score, where a thicker line indicates a higher interaction score. The genes are color-coded by their clustering, as determined by the Markov cluster algorithm score set to 3.0. The minimum interaction threshold used in this study was set to 0.700. The inputs used for the STRING database were the rice gene IDs.

### 4.10. RNA-seq and Data Analysis

WXS (S) were also grown in the paddy fields of Wuhan University, from July to August 2017, and WXS (F) was grown in the growth chambers, which was set an average temperature of 22 °C, and a 14-h light/10-h dark photoperiod and 77% relative humidity, and other environment factors close to the natural condition. The young panicles of WXS (S) and WXS (F) were harvested in P2 and P3, respectively. Total RNA was extracted from the young panicles using RNAiso Plus (#9108, TAKARA). Strand-specific RNA-seq libraries were synthesized using the NEBNextR UltraTM Directional RNA Library Prep Kit for Illumina R (NEB, Ipswich, MA, USA). The libraries were sequenced with the Illumina Hiseq Xten platform, and paired end reads were generated. The reads were aligned to the MSU7 reference by HISAT2, discarding low-quality alignments [48]. Gene expression levels were calculated using StringTie by counting the numbers of reads mapped to each gene [49]. Three biological replicates per sample were averaged for the differential expression analysis. Differential expression analysis of two samples was performed using the DESeq R package [50]. Genes with an expression fold-change >2 with a *p*-value < 0.05 were assigned as differentially expressed. The data were deposited in the Sequence Read Archive (SRA) at the National Center for Biotechnology Information (NCBI) under the accession number GSE.

### 4.11. RNA Isolation and Real Time RT-PCR

The total RNA was extracted from the samples by using the Trizol reagent (TaKaRa, Dalian, China) and was treated with DNase I (Fermentas, USA) to remove DNA contamination. In total, 2 μg RNA sample was used for reverse transcription with the RevertAid First Strand cDNA Synthesis Kit (Fermentas, USA) according to the manufacturer’s instructions. Quantitative real-time PCR (qPCR) was performed with a StepOne Plus real-time PCR system (Applied Biosystems) using a SYBR Green Master Mix (Roche, Germany). The primers used for the qPCR experiments are listed in Table S8. The qPCR reactions were implemented with three biological replicates. Actin was quantified as the internal control, and the relative expression were calculated using the 2^−ΔΔCt^ method [51].

## 5. Conclusions

In conclusion, we investigated the genetic and molecular mechanisms of fertility transition in the PTGMS rice line WXS by combining the results of proteomics that were obtained by iTRAQ with our transcriptomics results. We identified 340 differential expressed proteins (DAPs) and found that 196 DAPs were upregulated, and 144 DAPs were down regulated in WXS(S) and WXS (F), respectively. Among the DAPs and DEGs, 65 (SP2 vs. FP2) and 55 (SP3 vs. FP3) cor-DEGs-DAPs showed the same expression trend, and cor-DEGs-DAPs proteins were related to the energy metabolism-related proteins and to the activation of the stress response pathway and modifications to the cell wall, which ultimately affected the fertility transition of the PTGMS rice line WXS in anther development. We proposed a model for the protein regulation of PTGMS rice during the fertility transition stage, and the model showed that complicated network regulation leads to fertility transition in PTGMS rice.

## Figures and Tables

**Table 1:** The cor-DEGs-DAPs genes enriched in the cell wall GO term.

Accession	Description	Protein Fold Change		*p*-Value
		SP2/FP2	SP3/FP3	
*LOC_Os01g24680.1*	3-hydroxyacyl-CoA dehydrogenase, putative, expressed	−	0.81	1.0 × 10 ^−4^
*LOC_Os06g48180.1*	glycosyl hydrolases family 16 (GH16), putative, expressed	0.51	−	8.0 × 10 ^−4^
*LOC_Os05g04380.1*	peroxidase precursor, putative, expressed	0.51	−	1.0 × 10 ^−4^
*LOC_Os03g48770.1*	Cupin domain containing protein, expressed	0.48	−	9.0 × 10 ^−4^
*LOC_Os02g32980.1*	Cupin domain containing protein, expressed	0.42	−	1.0 × 10 ^−4^
*LOC_Os06g43640.1*	Ser/Thr protein phosphatase family protein, putative, expressed	−	0.74	2.1 × 10 ^−3^
*LOC_Os01g18170.1*	Cupin domain containing protein, expressed	0.69	−	1.9 × 10 ^−2^

**Table 2:** The cor-DEGs-DAPs proteins involved in protein processing in the endoplasmic reticulum.

Protein ID	Description	Protein Fold Change		*p*-Value
		SP2/FP2	SP3/FP3	
LOC_Os01g08860.1	hsp20/alpha crystallin family protein, putative, expressed	3.25	-	1.0 × 10 ^−4^
LOC_Os02g52150.2	heat shock 22 kDa protein, mitochondrial precursor, putative, expressed	2.19	-	2.0 × 10 ^−4^
LOC_Os03g60620.1	DnaK family protein, putative, expressed	0.7	-	1.0 × 10 ^−4^
LOC_Os04g01740.1	heat shock protein, putative, expressed	2.45	-	1.0 × 10 ^−4^
LOC_Os05g23740.1	DnaK family protein, putative, expressed	1.22	-	1.0 × 10 ^−4^
LOC_Os05g38530.1	DnaK family protein, putative, expressed	2.89	-	1.0 × 10 ^−4^
LOC_Os12g32986.1	heat shock protein, putative, expressed	1.4	-	1.0 × 10 ^−4^
LOC_Os03g45340.1	hsp20/alpha crystallin family protein, putative, expressed	−	0.74	2.4 × 10 ^−3^

## Supplementary Materials

Supplementary Materials can be found at https://www.mdpi.com/1422-0067/20/18/4542/s1. Figure S1 Experimental strategy for iTRAQ-based proteomic analysis of Wuxiang S (WXS). WXS (S): WXS plants were male sterile and cultivated in Wuhan; WXS (F): WXS plants were male fertility and cultivated in Hainan; P2: the PMC formation stage; P3: the meiosis stage of PMC. Three biological replicates were performed in this study. Figure S2 Venn diagram showing the numbers of all proteins identified by iTRAQ in three biological replicates. Figure S3 Protein mass (A), peptide length (B) and unique peptide number (C) in three biological replicates. (A): *X*-axis: Molecular weight (kDa); *Y*-axis: the corresponding protein number. (B): *X*-axis: the peptide length; *Y*-axis: the corresponding peptide percentage). (C): *X*-axis: the unique peptide number; *Y*-axis: the corresponding protein number). Figure S4 Classification of all of the identified proteins by COG, according to the protein function in plant metabolism. Figure S5 Overview of differentially abundant proteins (DAPs) in SP2 vs. FP2 and SP3 vs. FP3. Figure S6 GO categorization of all DAPs between in SP and FP. The GO analysis for gene functional classification shows the number of genes involved in the biological process, cellular component and molecular function. Figure S7 KEGG pathway analysis of all DAPs between WXS (S) and WXS (F) in two development stage (P2 and P3). Figure S8 Comparison of the expression ratios from transcriptomic (*y*-axis) and proteomic (*x*-axis) profiling based on the quantitative proteins and correlated genes of each compared group. Figure S9 Venn diagram showing the DAPs and DEGs between SP2 vs. SP3 (A) and FP2 vs. FP3 (B), SP2 vs. FP2 (C) and SP3 vs. FP3 (D). Figure S10 GO categorization of cor-DEGs-DAPs genes in SP2/FP2 and SP3/FP3. GO analysis for gene functional classification shows the number of genes involved in the biological process, cellular component and molecular function. Figure S11 The cor-DEGs-DAPs genes enriched in the cell wall of GO term (A) the cell wall synthesis pathway in the cellular component (GO:). (B) in the biological process (GO:). The cor-DEGs-DAPs genes are in green box. Figure S12 KEGG pathway enrichment analysis maps of protein processing in endoplasmic reticulum. The proteins in different red frames are cor-DAPs-DEGs identified in this study. The box represents proteins; the arrow represents activation. Figure S13 KEGG pathway enrichment analysis maps of starch and sucrose metabolism. The proteins in different green frames are cor-DAPs-DEGs identified in this study. The box represents proteins; the arrow represents activation. Figure S14 KEGG pathway enrichment analysis maps of Cutin, suberine and wax biosynthesis. The proteins in different green frames are cor-DAPs-DEGs identified in this study. The box represents proteins; the arrow represents activation.

## Abbreviations

PTGMS	photo-thermosensitive genic male sterile
DEP	differentially expressed gene
DAP	differentially abundant protein
cor-DEGs-DAPs	corresponding DEGs and DAPs
qRT-PCR	quantitative RT-PCR
PMC	pollen mother cell
P2	the PMC formation stage
P3	the meiosis stage of PMC
