# Supplementary material for: Comparative Analysis of Proteomics and Transcriptomics during Fertility Transition in a Two-Line Hybrid Rice Line Wuxiang S
Source: Int J Mol Sci. 2019 Sep 13;20(18):4542. doi: 10.3390/ijms20184542 (PMC6770272; doi:10.3390/ijms20184542)
Supplement: Supplementary file 1 [file ijms-20-04542-s001.zip › Table S1.docx]

**Table S1 Overview of protein identification**

| Sample name | Total spectra | Unique spectra | Unique peptide | Protein |
| --- | --- | --- | --- | --- |
| Replication 1 | 534811 | 110277 | 42710 | 5671 |
| Replication 2 | 536849 | 113684 | 46000 | 6470 |
| Replication 3 | 538020 | 116816 | 43073 | 6005 |
