# Supplementary material for: Comparative Analysis of Proteomics and Transcriptomics during Fertility Transition in a Two-Line Hybrid Rice Line Wuxiang S
Source: Int J Mol Sci. 2019 Sep 13;20(18):4542. doi: 10.3390/ijms20184542 (PMC6770272; doi:10.3390/ijms20184542)
Supplement: Supplementary file 1 [file ijms-20-04542-s001.zip › Table S8.docx]

| **Primer name** | **ID** | **Primer sequences (5'-3')** |
| --- | --- | --- |
| MAS-F | LOC_Os04g40990.1 | GACTTCGGCCTCTACTTCTTC |
| MAS-R |  | TCCCTAGAGTGCTCCATCTT |
| GDSL-F | LOC_Os05g11910.1 | CAGGGTCATGTACGCTGATTT |
| GDSL-R |  | CACACCTTCAGCCCGTATTT |
| Eno-F | LOC_Os01g54860.1 | CTCCTGTCTCGAGGTTGTTTAC |
| Eno-R |  | CCACCCATGACAAGTCCATTA |
| SSRP-F | LOC_Os05g08970.1 | GCCACCACTTCAACAACATATC |
| SSRP-R |  | ATGCAAGTCCTCCCGAATAAA |
| KET-F | LOC_Os02g57260.1 | GGGTTCTACGACATAGGGATTG |
| KET-R |  | CTGTGCTTTCTGGACTTCATTTAC |
| SALT-F | LOC_Os02g18410.1 | GCGAGTTCGAGGAGAAGAAG |
| SALT-R |  | CTGTCCTTGATCAGAACCTTGA |
| GSC-F | LOC_Os02g50240.1 | GACAGTGGGAGTTCCAAGTT |
| GSC-R |  | CTCGGTGATCCTCTCAAGAATG |
| Hsp22-F | LOC_Os02g52150.2 | GATCAAGGCGGAGATGAAGAA |
| Hsp22-R |  | CGACGTTGACCTGGAAGAC |
| Actin-F |  | GATCACTGCCTTGGCTCCTA |
| Actin-R |  | GTACTCAGCCTTGGCAATCC |

**Table S8 Primers for qRT-PCR.**
