# Supplementary figures and images for: Comparative Analysis of Proteomics and Transcriptomics during Fertility Transition in a Two-Line Hybrid Rice Line Wuxiang S
Source: Int J Mol Sci. 2019 Sep 13;20(18):4542. doi: 10.3390/ijms20184542 (PMC6770272; doi:10.3390/ijms20184542)

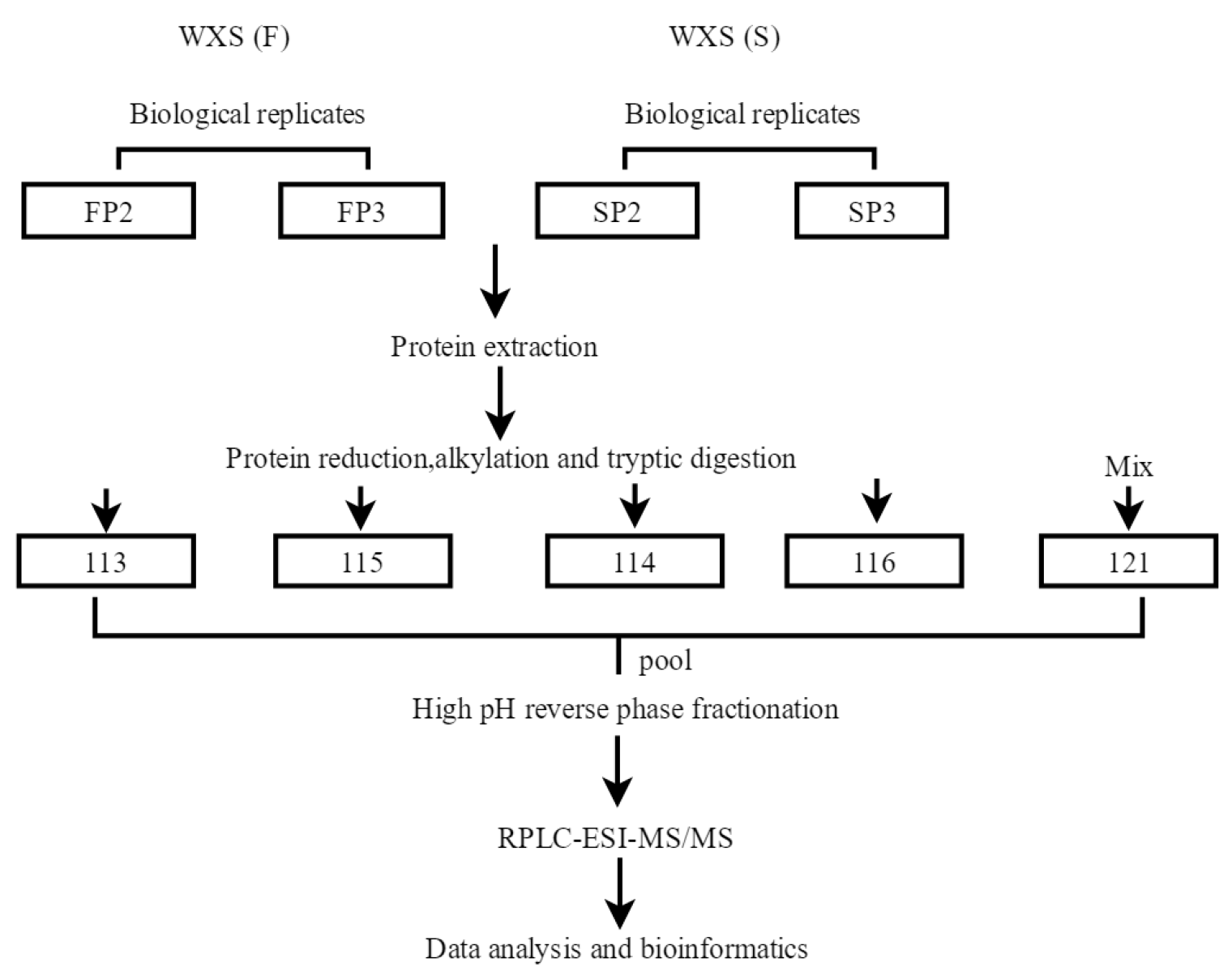

Supplement: Supplementary file 1 [file ijms-20-04542-s001.zip › Figure S1.tif]

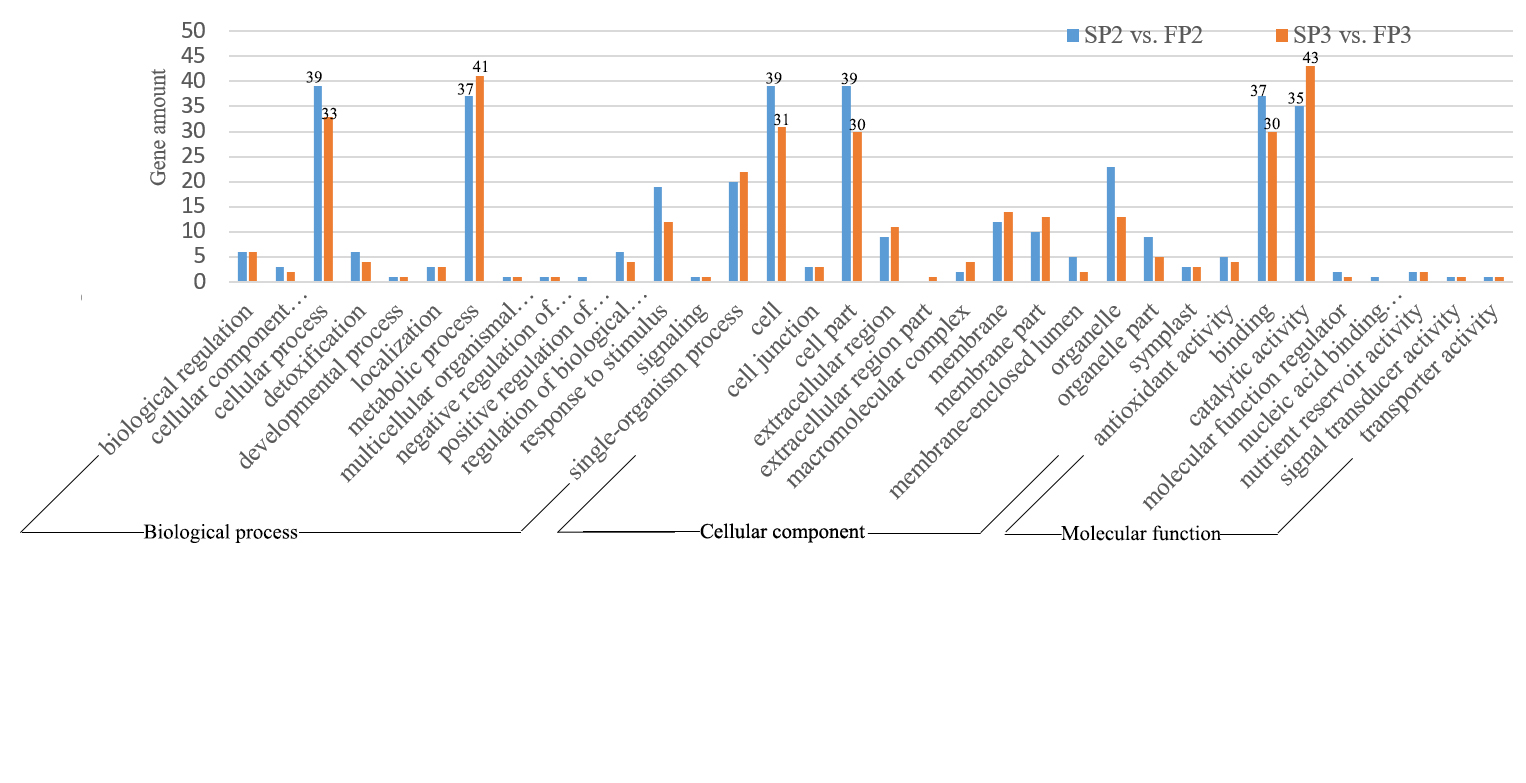

Supplement: Supplementary file 1 [file ijms-20-04542-s001.zip › Figure S10.tif]

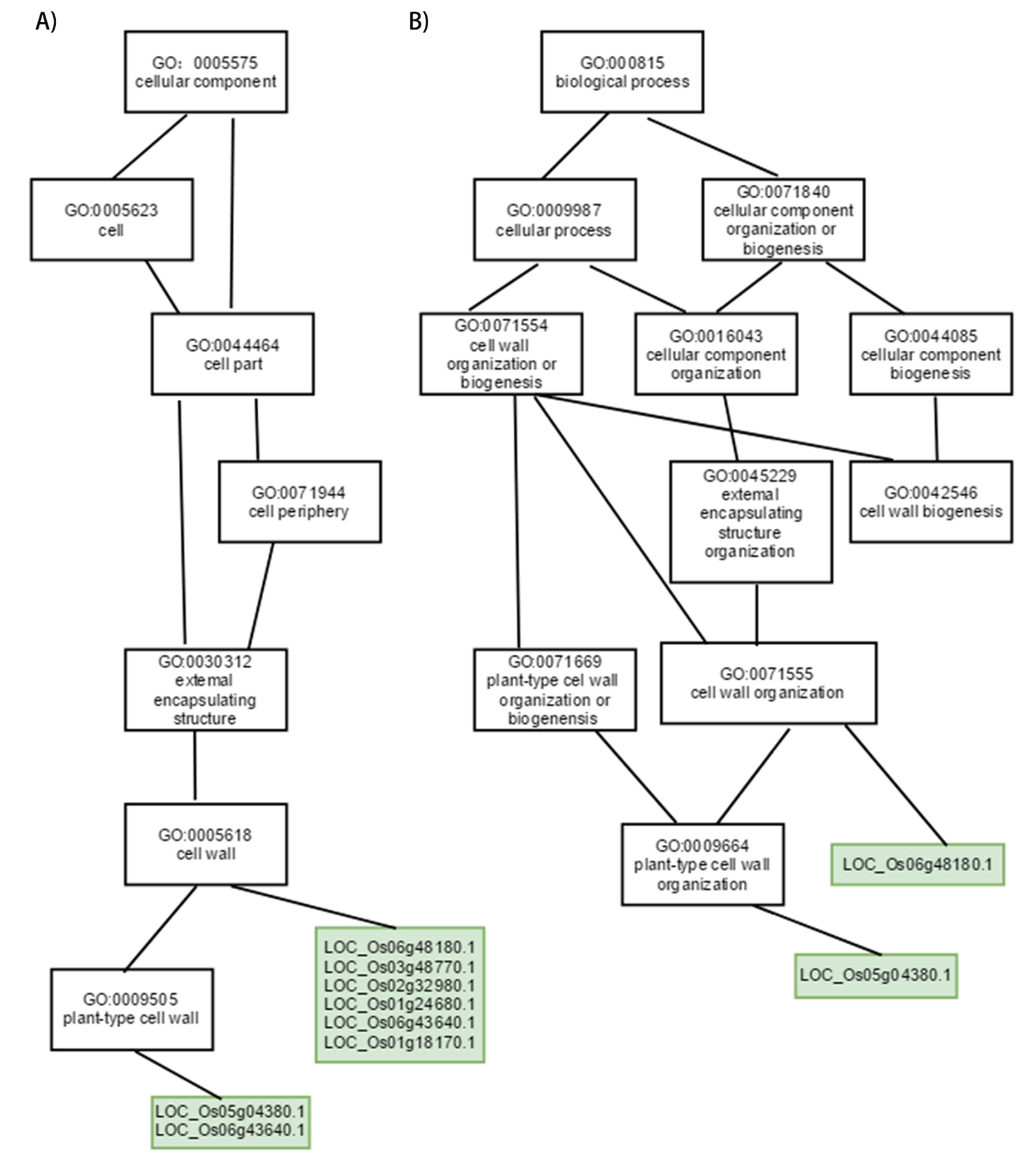

Supplement: Supplementary file 1 [file ijms-20-04542-s001.zip › Figure S11.tif]

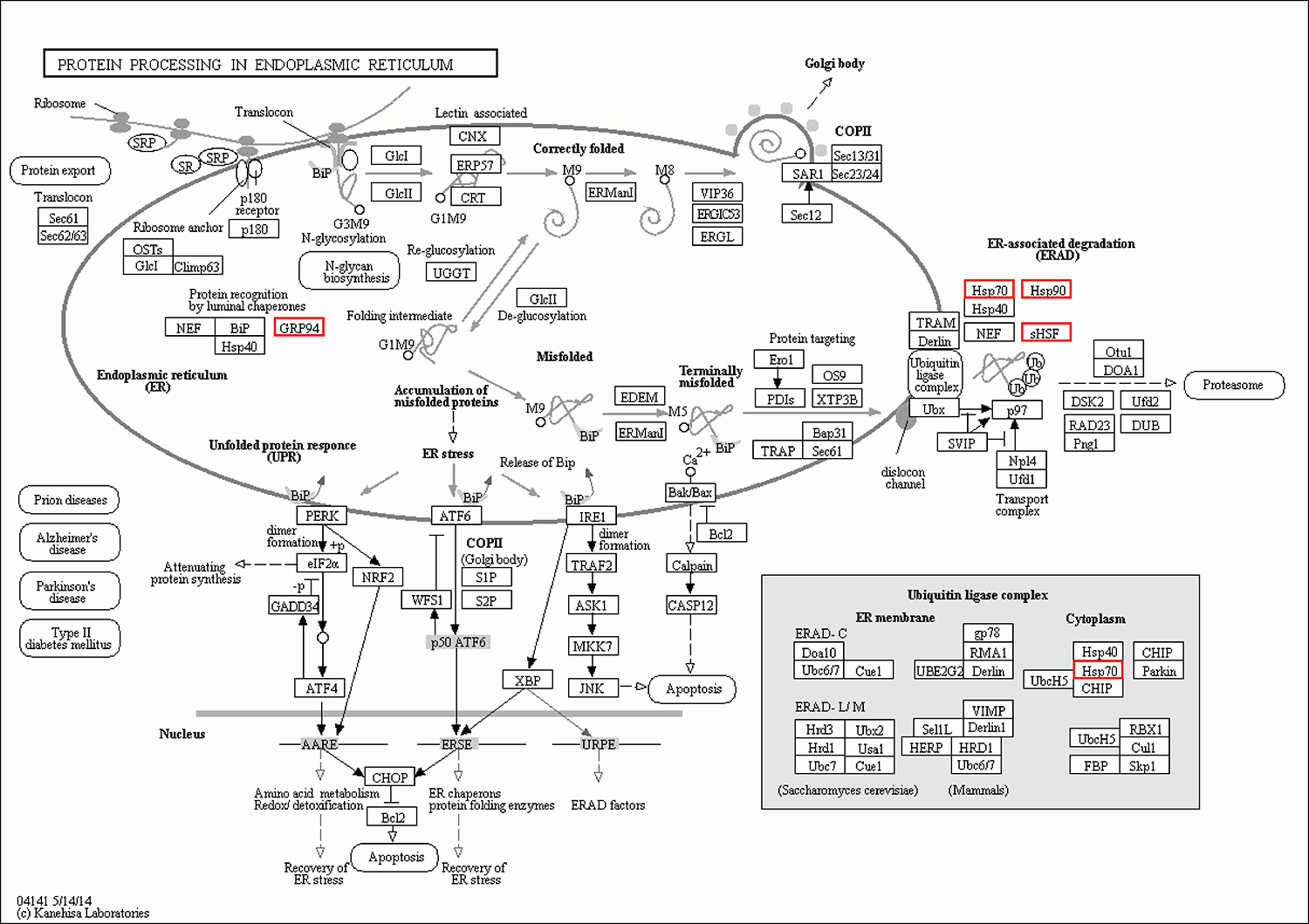

Supplement: Supplementary file 1 [file ijms-20-04542-s001.zip › Figure S12.tif]

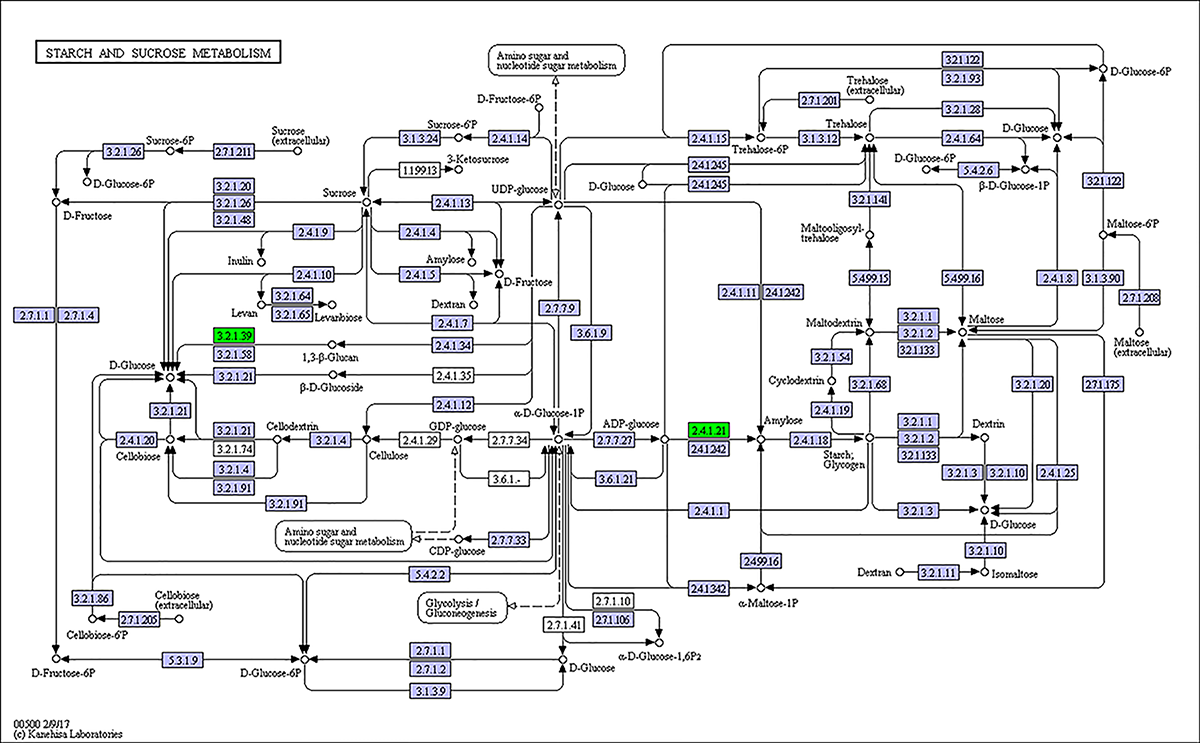

Supplement: Supplementary file 1 [file ijms-20-04542-s001.zip › Figure S13.tif]

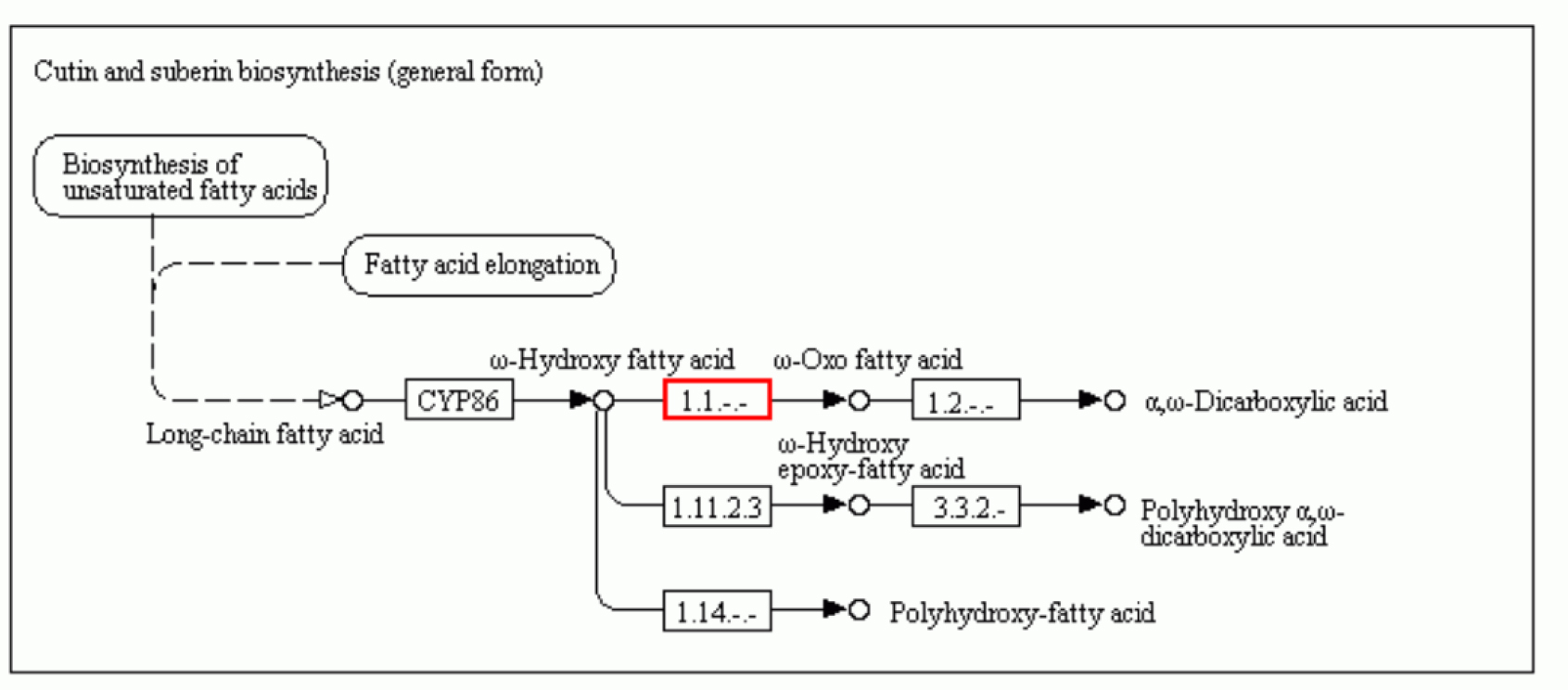

Supplement: Supplementary file 1 [file ijms-20-04542-s001.zip › Figure S14.tif]

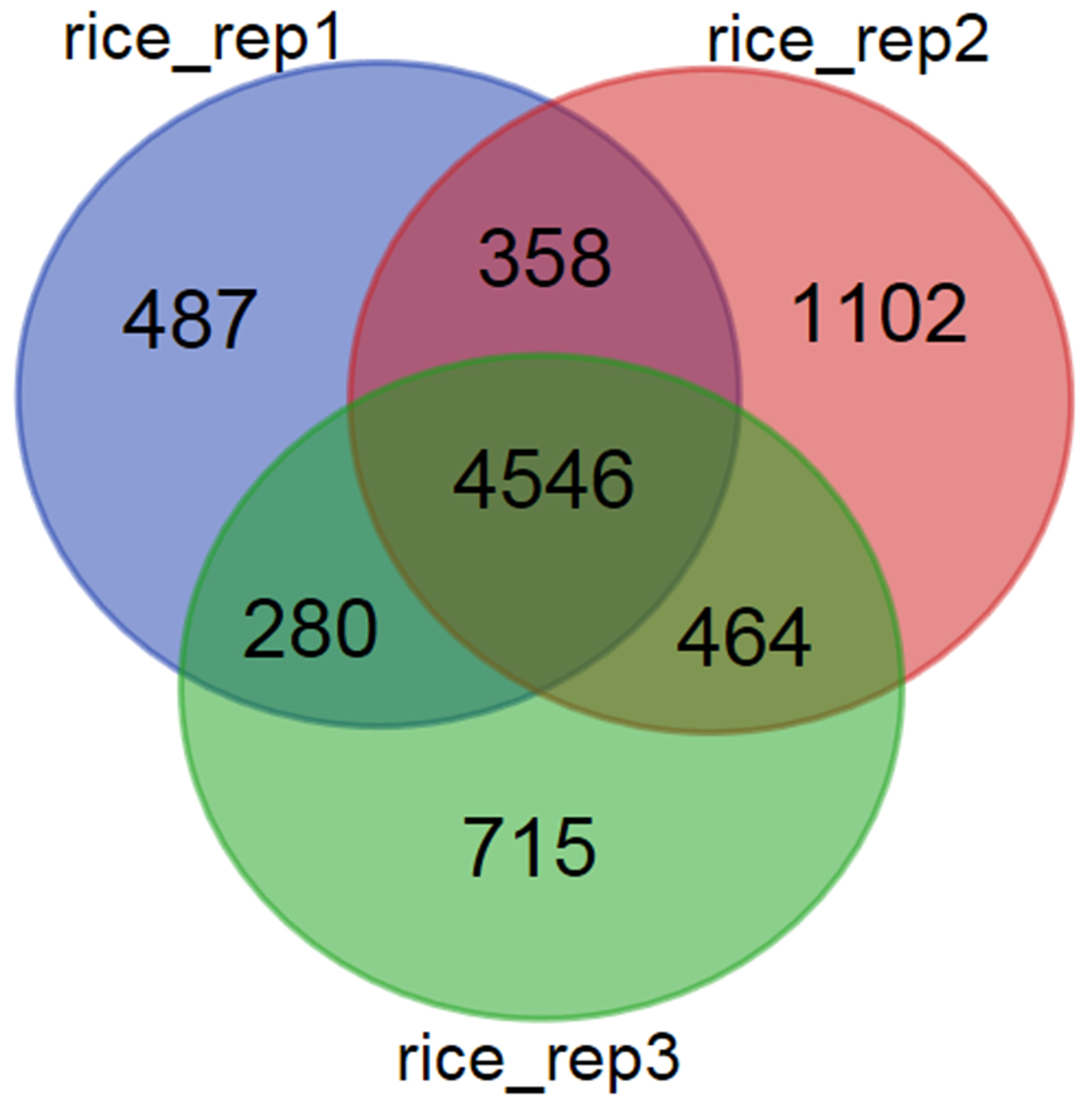

Supplement: Supplementary file 1 [file ijms-20-04542-s001.zip › Figure S2.tif]

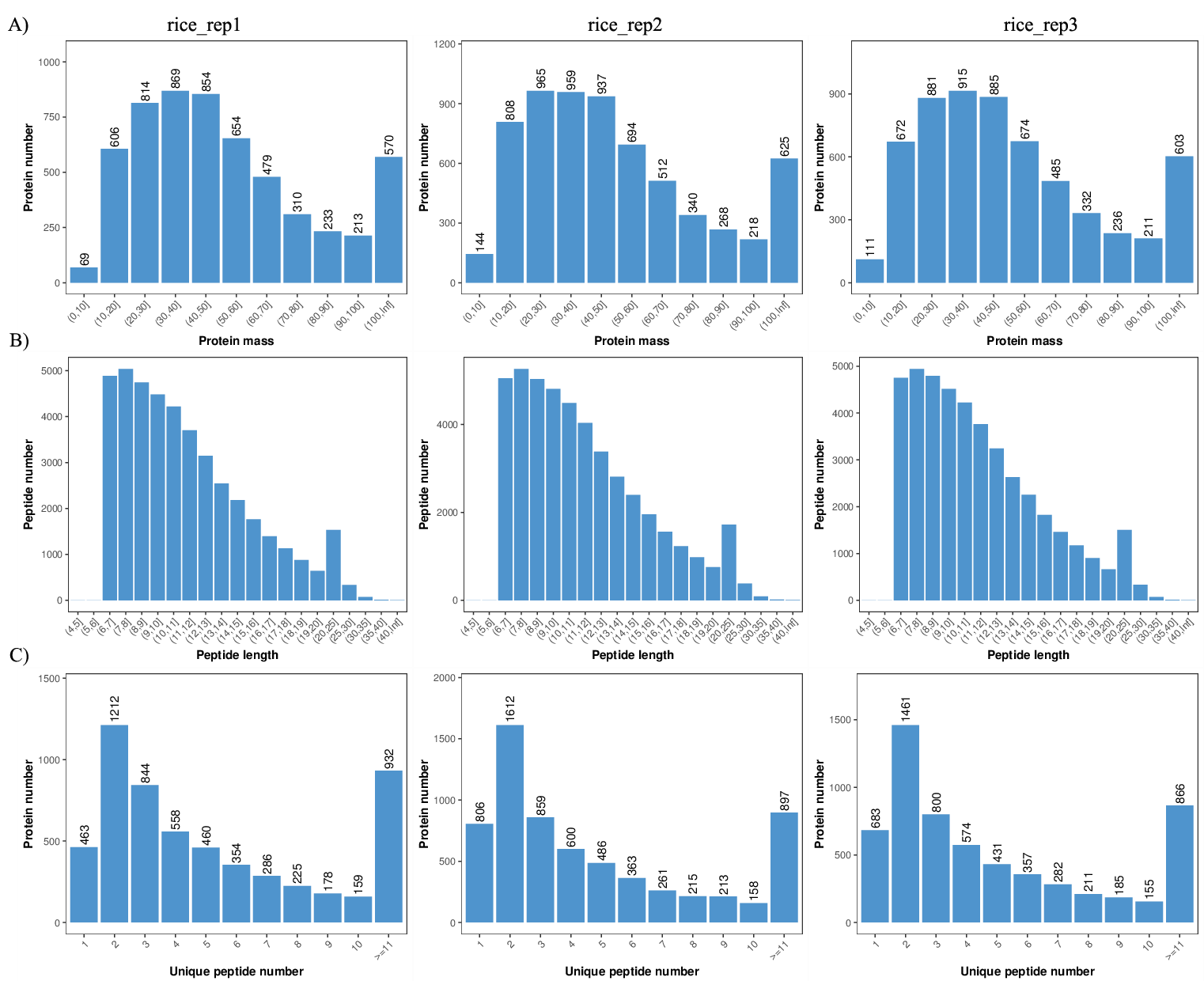

Supplement: Supplementary file 1 [file ijms-20-04542-s001.zip › Figure S3 .tif]

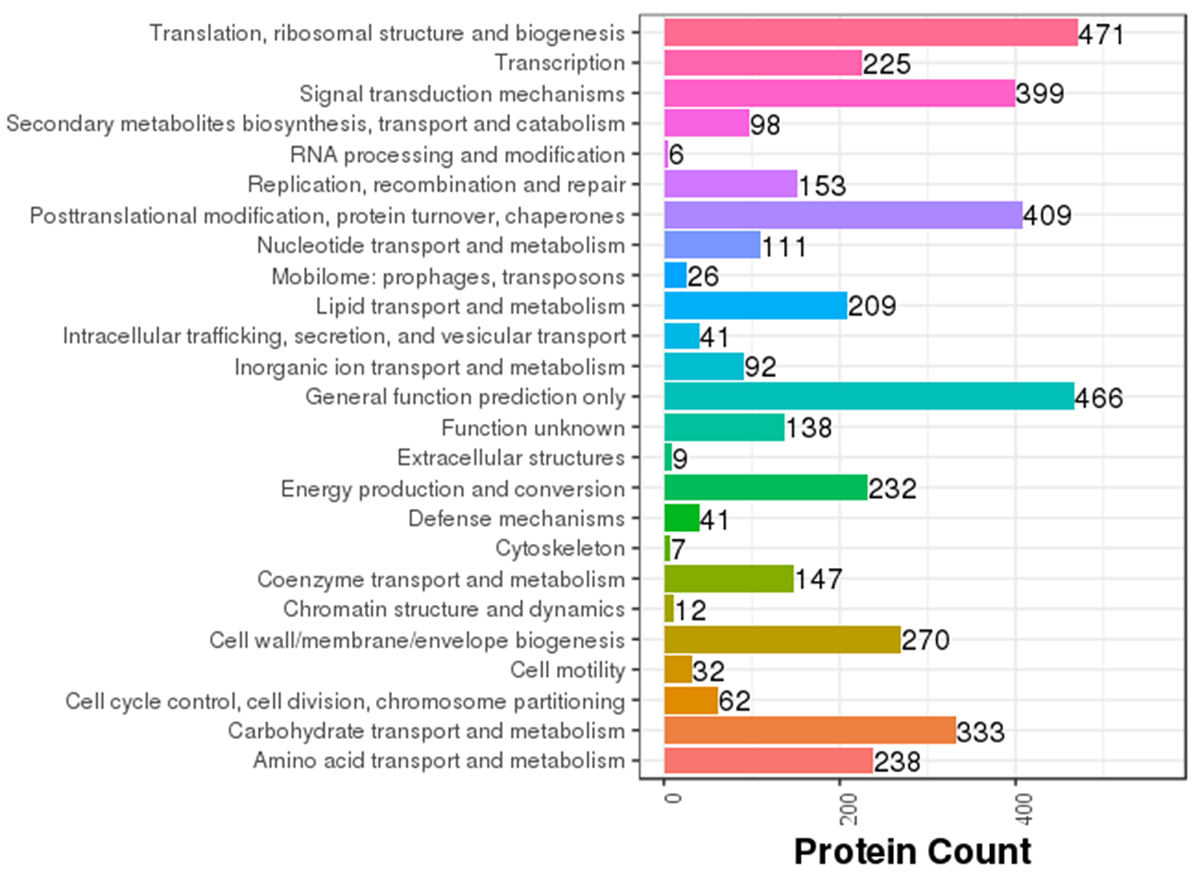

Supplement: Supplementary file 1 [file ijms-20-04542-s001.zip › Figure S4 .tif]

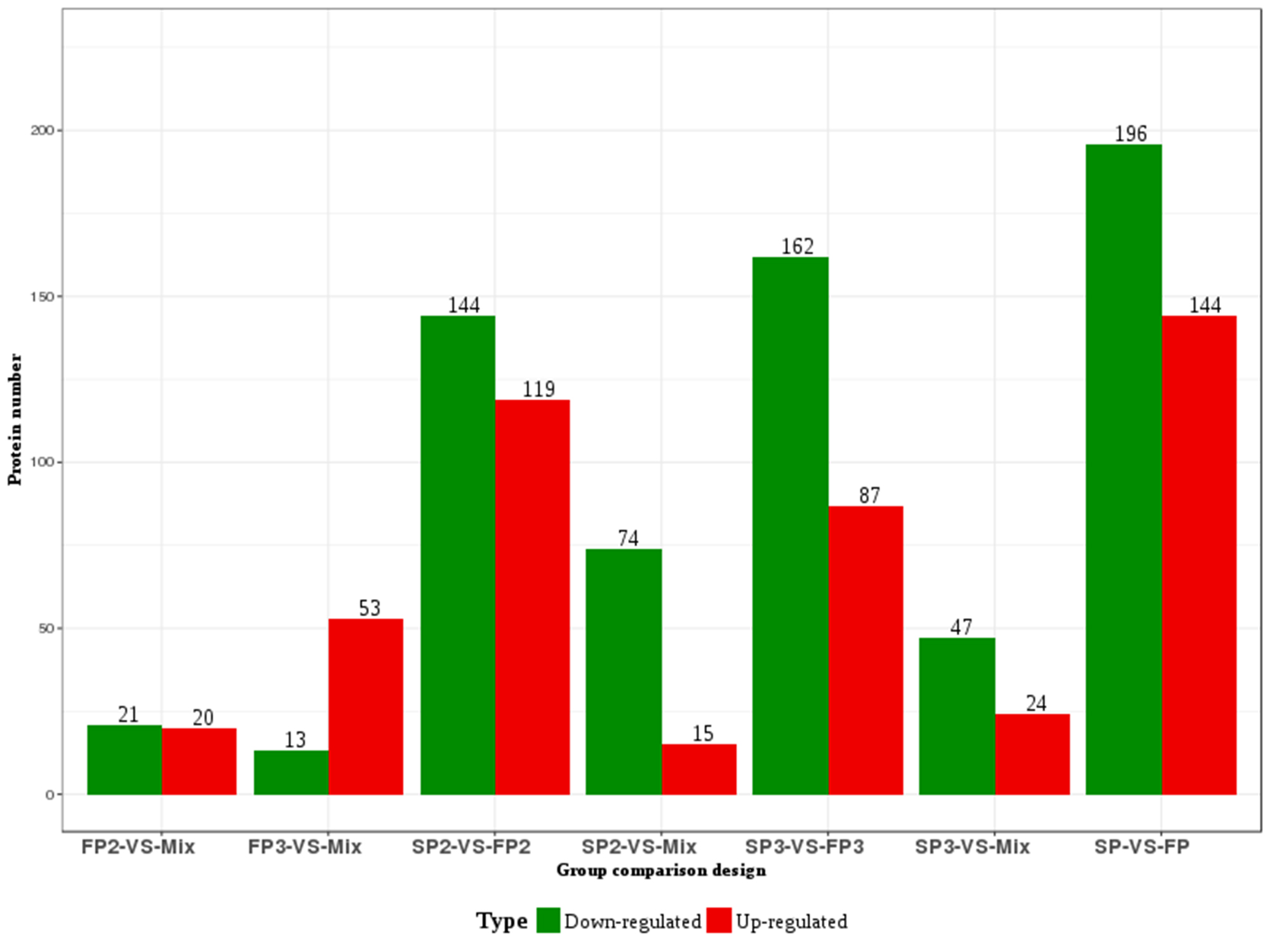

Supplement: Supplementary file 1 [file ijms-20-04542-s001.zip › Figure S5 .tif]

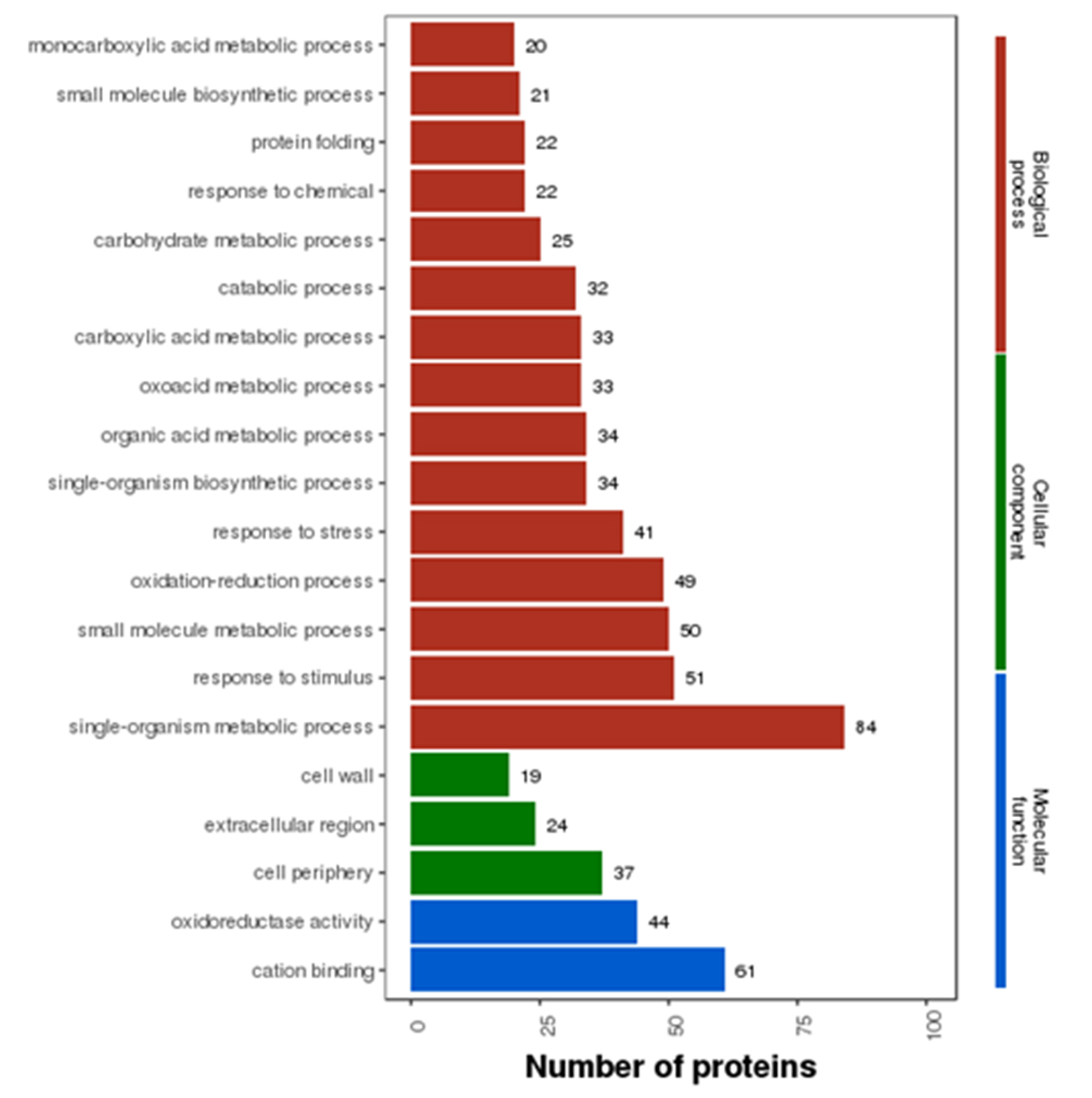

Supplement: Supplementary file 1 [file ijms-20-04542-s001.zip › Figure S6 .tif]

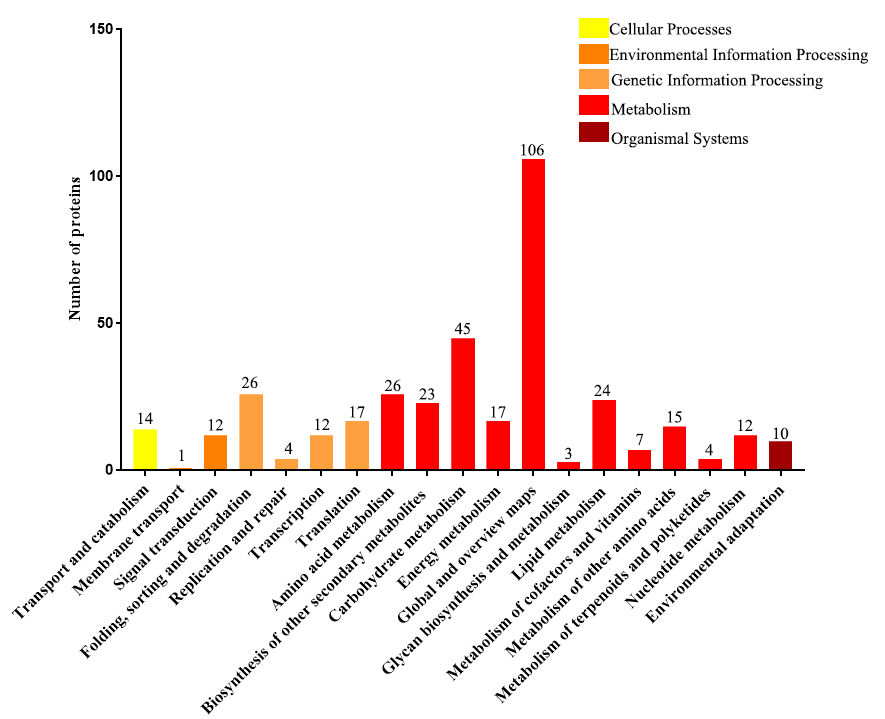

Supplement: Supplementary file 1 [file ijms-20-04542-s001.zip › Figure S7.tif]

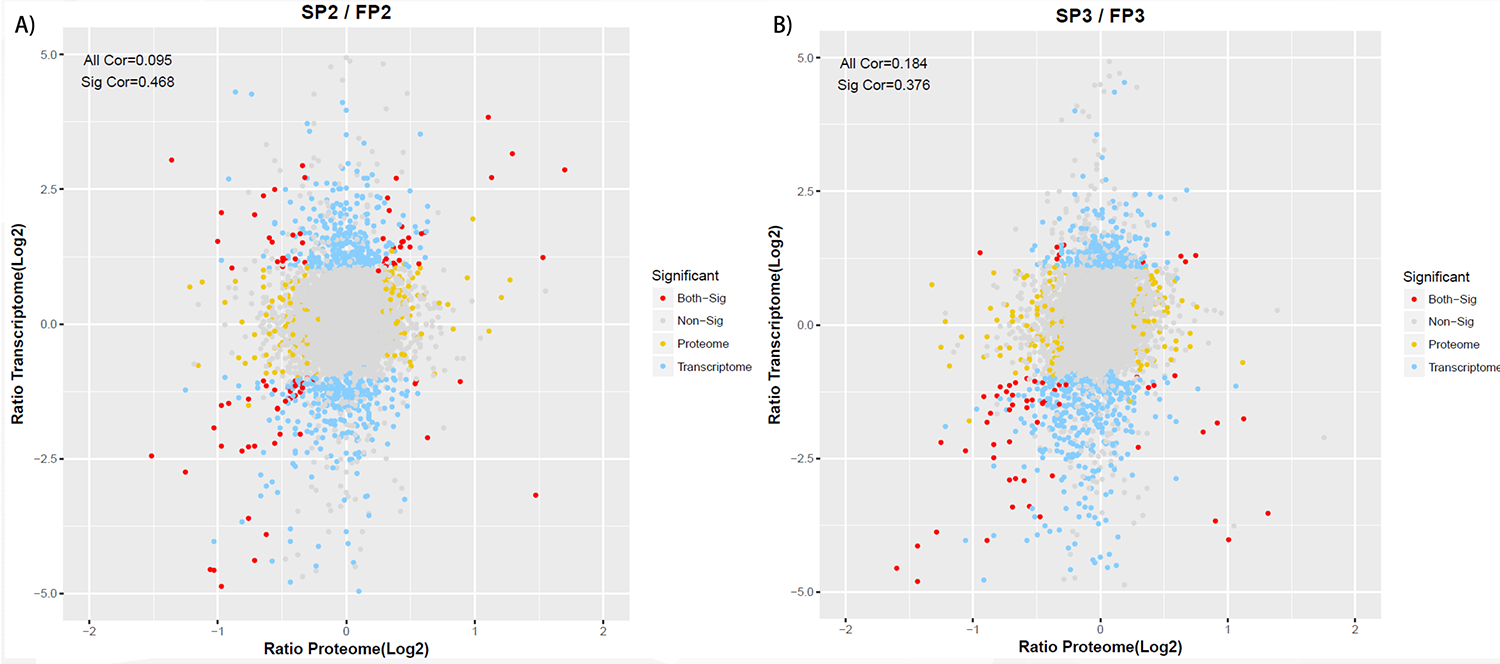

Supplement: Supplementary file 1 [file ijms-20-04542-s001.zip › Figure S8.tif]

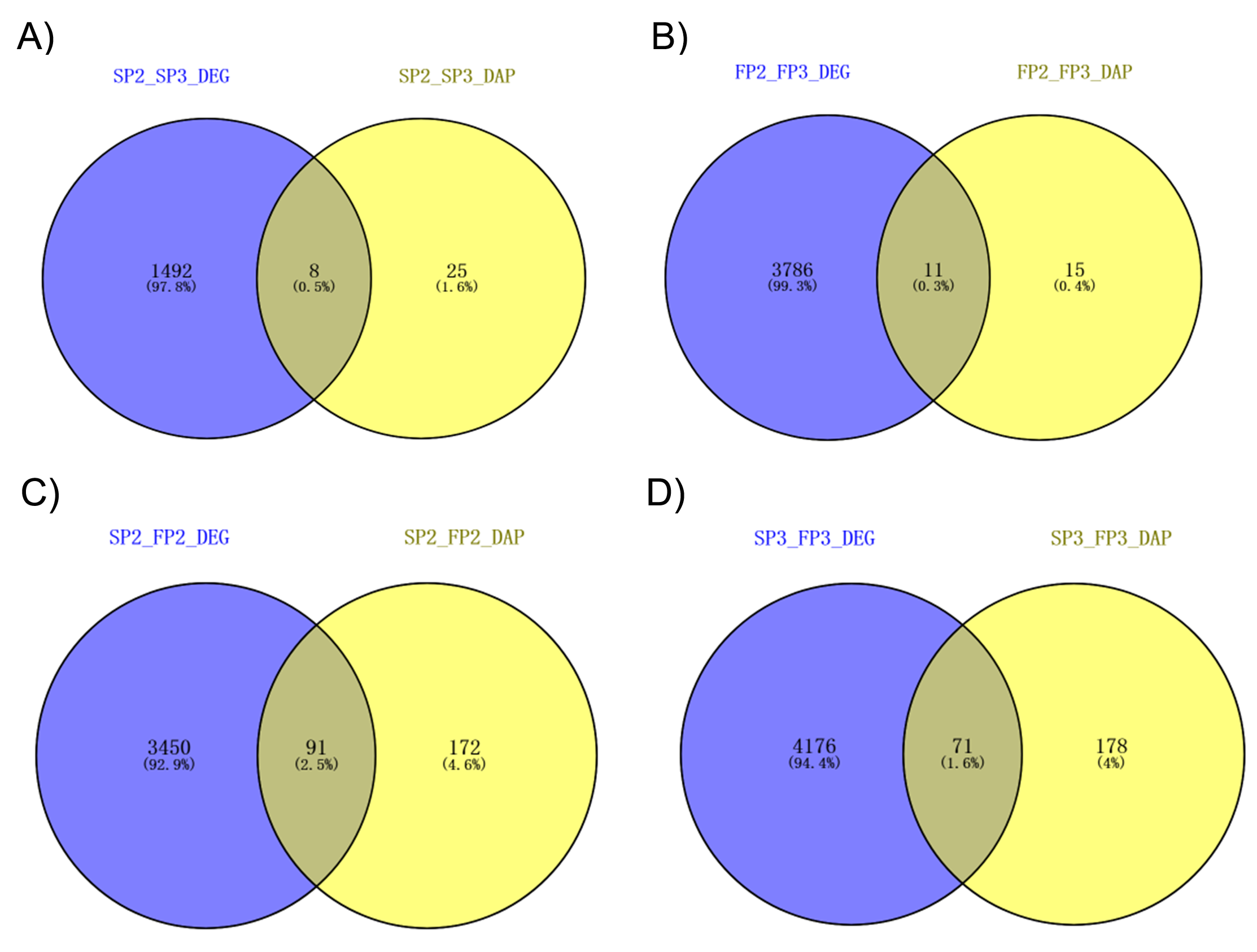

Supplement: Supplementary file 1 [file ijms-20-04542-s001.zip › Figure S9.tif]
